# Prognostic value of neurofilament light chain in chronic inflammatory demyelinating polyneuropathy

**DOI:** 10.1093/braincomms/fcab018

**Published:** 2021-03-09

**Authors:** Joris Godelaine, Maxim De Schaepdryver, Xavier Bossuyt, Philip Van Damme, Kristl G Claeys, Koen Poesen

**Affiliations:** Department of Neurosciences, Laboratory for Molecular Neurobiomarker Research, Leuven Brain Institute, KU Leuven, Leuven 3000, Belgium; Department of Laboratory Medicine, University Hospitals Leuven, Leuven 3000, Belgium; Department of Neurosciences, Laboratory for Molecular Neurobiomarker Research, Leuven Brain Institute, KU Leuven, Leuven 3000, Belgium; Department of Laboratory Medicine, University Hospitals Leuven, Leuven 3000, Belgium; Department of Microbiology, Immunology and Transplantation, Clinical and Diagnostic Immunology, KU Leuven, Leuven 3000, Belgium; Department of Neurology, University Hospitals Leuven, Leuven 3000, Belgium; Department of Neurosciences, Experimental Neurology, Laboratory of Neurobiology, Leuven Brain Institute, VIB KU Leuven Center for Brain and Disease Research, Leuven 3000, Belgium; Department of Neurology, University Hospitals Leuven, Leuven 3000, Belgium; Department of Neurosciences, Laboratory for Muscle Diseases and Neuropathies, Leuven Brain Institute, KU Leuven, Leuven 3000, Belgium; Department of Neurosciences, Laboratory for Molecular Neurobiomarker Research, Leuven Brain Institute, KU Leuven, Leuven 3000, Belgium; Department of Laboratory Medicine, University Hospitals Leuven, Leuven 3000, Belgium

**Keywords:** CIDP, neurofilament light chain, biomarkers, prognosis

## Abstract

Chronic inflammatory demyelinating polyneuropathy is a neuroinflammatory disorder with considerable variation in clinical phenotype, disease progression and therapy response among patients. Recently, paranodal antibodies associated with poor response to intravenous immunoglobulin therapy and more aggressive disease course have been described in small subsets of patients, but reliable serum-based prognostic biomarkers are not yet available for the general population. In current retrospective longitudinal study, we utilized logistic regression models to investigate the associations of serum neurofilament light chain levels with 1-year disease progression and therapy response during follow-up in chronic inflammatory demyelinating polyneuropathy. One-year disease progression was defined as a decrease of four or more points (the minimal clinically important difference) on an 80-point Medical Research Council sum-score scale 1 year after sampling. Patients who, compared to treatment received at time of sampling, required therapy switch during follow-up due to insufficient effect were classified as non-responders. Serum neurofilament light chain was measured by electrochemiluminescence assay in clinical residual serum samples of 76 patients diagnosed with probable (13 patients) or definite (63 patients) chronic inflammatory demyelinating polyneuropathy according to European Federation of Neurological Societies/Peripheral Nerve Society diagnostic criteria. Eleven (15%) patients were female, and the mean (standard deviation) cohort age was 61.5 (11.7) years. In both univariate and multivariable (including demographics) models, elevated serum neurofilament light chain harboured increased odds for 1-year disease progression (respectively odds ratio, 1.049; 95% confidence interval, 1.022–1.084 and odds ratio, 1.097; 95% confidence interval, 1.045–1.169; both *P* *=* 0.001). Patients with levels above the median cohort neurofilament light chain level (28.3 pg/ml) had largely increased odds of 1-year disease progression (univariate: odds ratio, 5.597; 95% confidence interval, 1.590–26.457; *P* = 0.01; multivariable: odds ratio, 6.572; 95% confidence interval, 1.495–39.702; *P* = 0.02) and of insufficient treatment response (univariate: odds ratio, 4.800; 95% confidence interval, 1.622–16.442; *P* = 0.007; multivariable: odds ratio, 6.441; 95% confidence interval, 1.749–29.357; *P* = 0.009). In a combined approach analysis, patients with levels above median cohort serum neurofilament light chain level reported strongly increased odds of demonstrating 1-year disease progression and/or therapy non-response during follow-up (univariate: odds ratio, 6.337; 95% confidence interval, 2.276–19.469; *P* < 0.001; multivariable: odds ratio, 10.138; 95% confidence interval, 2.801–46.404; *P* *=* 0.001). These results show that in various logistic regression models, serum neurofilament light chain was associated with both 1-year disease progression and therapy response during follow-up in chronic inflammatory demyelinating polyneuropathy. Hence, our findings warrant further prospective research regarding the value of neurofilament light chain as potential prognostic biomarker in chronic inflammatory demyelinating polyneuropathy.

## Teaser

Godelaine et al. investigated the prognostic value of serum neurofilament light chain in chronic inflammatory demyelinating polyneuropathy and showed in various logistic regression models that elevated serum neurofilament light chain levels were significantly associated with increased odds of 1-year disease progression, not responding to therapy during follow-up or both.

## Graphical Abstract

**Graphical Abstract:**
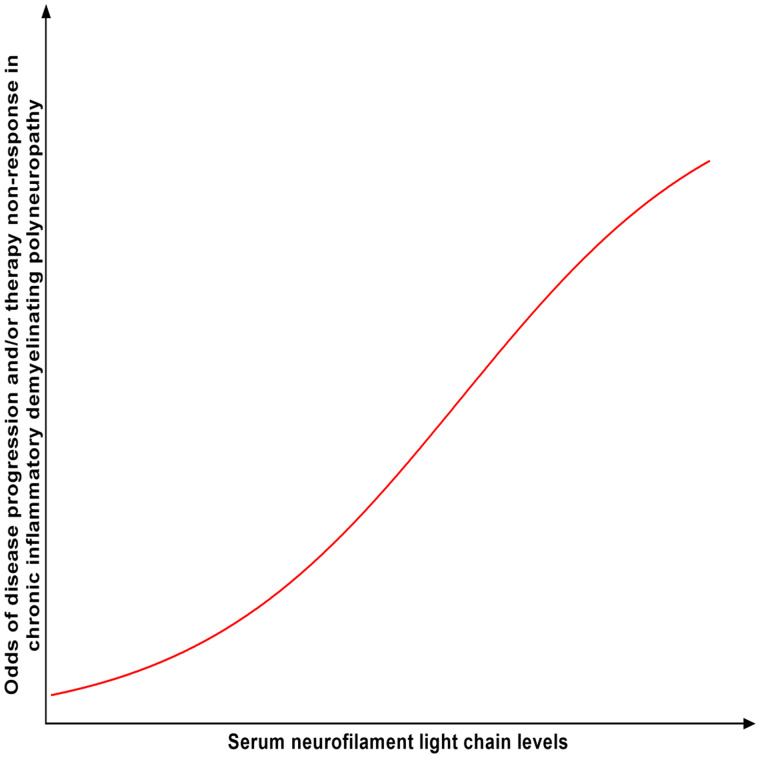

## Introduction

Chronic inflammatory demyelinating polyneuropathy (CIDP) demonstrates considerable variation in clinical phenotype, disease progression and therapy response among patients.^1–3^ While some patients remain stable for years with no or only minimal immunomodulatory therapy, others progress more rapidly or experience lasting disability despite intensive long-term treatment.^3–5^ However, it is not yet possible to accurately predict how individual patients will progress. Also, it is estimated that eventually up to 30% of patients will not respond well to one of CIDP’s main therapies (intravenous immunoglobulins, plasma exchange or steroids), necessitating therapy switch to halt or limit disease progression.^3–5^ Recently, paranodal autoantibodies associated with poor response to intravenous immunoglobulins and more aggressive disease course have been described in small subsets of CIDP patients.^6–8^ To this date, however, reliable prognostic serum-based biomarkers applicable to the general population of CIDP patients are not yet available.^9^

In various neurological diseases, blood-based biomarkers that reflect disease progression have recently been gaining attention, including serum neurofilament light chain (sNfL).^10–25^ Neurofilaments are released into blood and cerebrospinal fluid of patients with neurodegenerative diseases in whom they have been shown to be associated with various clinical features.^10–25^ For example, recent studies showed significant cross-sectional associations between sNfL and disability or therapy response in various peripheral neuropathies, including in CIDP.^19–23^ However, these studies did not yet investigate the prognostic value of sNfL in CIDP with respect to disease progression or therapy response. As such, this retrospective longitudinal study aimed to investigate the prognostic value of sNfL levels in CIDP patients with respect to 1-year disease progression and therapy response during follow-up.

## Materials and methods

### Patient inclusion

For this single-centre retrospective longitudinal study, adult patients had to comply with following inclusion criteria: (1) having received a probable or definite CIDP diagnosis according to the European Federation of Neurological Societies/Peripheral Nerve Society diagnostic criteria by a trained neurologist; (2) having a clinical residual serum sample stored at the biobank of the University Hospitals Leuven from 2009 the earliest; (3) having been followed up at the University Hospitals Leuven NeuroMuscular Reference Center for at least one year after blood sampling; and (4) availability of demographic and clinical data in electronic medical records, including information on therapy response and/or disease progression. Patients who experienced around time of sampling any event that might influence sNfL levels (e.g. stroke, infection, documented head trauma, etc.) were not considered. All patients agreed with storage of residual material for future research by opting-out agreement. Ethical approval for the study was granted by Ethical Committee Research UZ/KU Leuven (S62265).

### Patient classification

Included patients were classified according to both 1-year disease progression (‘progressor’ or ‘non-progressor’) and therapy response during follow-up (‘responder’ or ‘non-responder’) via retrospective study of medical records:

One-year disease progression was defined as a decrease of at least four points (the minimal clinically important difference^26^^,^^27^) on an 80-point Medical Research Council (MRC) sum-score scale one year after serum sampling (assessed by consulting the medical record closest to one year after sampling) compared to the MRC sum-score measured at time of sampling (‘baseline’ MRC sum-score). The 80-point MRC sum-score, previously applied in clinical trials,^28^ is the sum of scores for eight bilateral (left and right side) muscle groups each rated between zero (no visible contraction) to five (normal movement) and is used at the University Hospitals Leuven NeuroMuscular Reference Center to follow-up patients. Higher scores indicate greater muscle contraction/limb movement.

Therapy response during follow-up was assessed for each patient as followed: We examined what treatment (intravenous immunoglobulins, plasma exchange, steroids, immunosuppressive drugs or a combination of therapies) was utilized at time of sampling (‘baseline’ treatment). When no therapy was yet initiated, the treatment established closest to time of sampling was designated baseline treatment. When during follow-up a therapy switch from this baseline treatment was not required (assessed by consulting all available medical records of the patient from time of sampling until the time of this study, with a minimum interval of one year between therapy initiation and last medical record evaluated), patients were classified as ‘therapy responders’. However, when a therapy switch was required due to insufficient treatment effect based on clinical judgement by a trained neurologist (e.g. based on a deterioration on disability/impairment scales, persisting pain, subjective patient feeling of general decline, etc.), patients were classified as ‘non-responders’. When therapy was switched for other reasons than insufficient treatment effect (e.g. side effects, practicality of administration, decrease in disease activity etc.) but the patient *did* respond to baseline therapy, the patient was still defined as ‘therapy responder’.

### sNfL measurements

Peripheral blood sampling and isolation of serum was performed according to standardized operating procedures during routine clinical practice in diagnostic work-up and follow-up of patients. As part of routine practice, residual material was stored in the biobank of the University Hospitals Leuven clinical laboratory at −20°C. For each patient included in the study, the most relevant serum sample available in the biobank (i.e. closest to diagnosis) was sorted out for sNfL measurement. sNfL was measured using a previously established electrochemiluminescence assay^29^: 96-well Multi-Array^®^ Standard plates (Meso Scale Discovery, Rockville, MD, USA) were coated using 30 µL capture antibody (mAb 47:3, 1.25 µg/ml; Uman Diagnostics, Umea, Sweden) diluted in TBS. All following steps were performed on a plate shaker (500 rpm) at room temperature and were preceded by three wash steps using TBS containing 0.1% Tween 20. Non-specific binding sites were blocked for one hour using 100 µl of 3% non-fat dried milk in TBS. After blocking, 25 µl of sample, blank or calibrator (lyophilized bovine NfL, range 7.8–1000 pg/ml; Uman Diagnostics) was added to each well followed by a 2-h incubation. After washing, 25 µl of detection antibody (mAb 2:1, 0.5 µg/ml; Uman Diagnostics), diluted in TBS containing 1% non-fat dried milk and 0.1% Tween 20, was added to each well and incubated for 1 h. Next, MSD SULFO-TAG™ labelled streptavidin (0.25 µg/ml), diluted in TBS containing 1% non-fat dried milk and 0.1% Tween 20, was added and incubated for 1 h. Following a final wash, 150 µl of 2× ECL read buffer (Meso Scale Discovery) was added and the ECL signal measured using a Meso Quickplex SQ120 multiplex imager (Meso Scale Discovery). A four-parameter weighted logistic fit curve was generated and sample concentrations extrapolated. Samples were measured in duplicate and all coefficients of variation of duplicate determinations were less than 10%. Results below the previously reported lower limit of quantification (LLOQ) of the assay (15.6 pg/ml)^30^ were not discarded or transformed to a variation in the LLOQ (e.g. LLOQ and LLOQ/√2) but were used as such to improve performance of statistical models.^31^

### Statistical analysis

Univariate and multivariable linear least square regression models were used to investigate associations between sNfL levels and demographics (age, disease duration, CIDP phenotype, sex) and MRC sum-score at time of sampling. Linear regression models, which demonstrated best model fit (in comparison with quadratic, cubic and logarithmic models), were also used to evaluate the association between sNfL levels and the change in MRC sum-score patients demonstrated over a 1-year period. Regression residuals showed no heteroscedasticity or important deviations from normality. sNfL levels were compared between 1-year disease progression groups (progressors versus non-progressors) and therapy response during follow-up groups (responders versus non-responders) using either Student’s *t*-tests or non-parametric Mann–Whitney *U* tests based on distribution of data as assessed by Shapiro–Wilk test.

Logistic regression models were used to investigate the association between sNfL levels and 1-year disease progression and therapy response during follow-up. Both univariate and multivariable logistic regression models were used, with multivariable models including the demographics age, disease duration, sex and CIDP phenotype (classical or atypical) to correct for demographic heterogeneity in the cohort. Patients with missing data regarding 1-year disease progression or therapy response during follow-up were excluded for the associated statistical analyses. In a combined approach, logistic regression models were also used to evaluate the ability of sNfL to differentiate patients who demonstrated either 1-year disease progression, therapy non-response during follow-up or both from patients who remained stable on MRC sum-score while also responding to baseline therapy (i.e. responding non-progressors). In all models, sNfL was evaluated both as continuous variable and as binary variable through stratification of patients as having low or high sNfL based on median cohort sNfL. Statistical analyses were performed using IBM SPSS (V26.0, IBM Corp., Armonk, NY, USA). α = 0.05 was used as cut-off for significance. As this was an exploratory study, no corrections for multiple comparisons were made.^32^^,^^33^ A power calculation could not be performed due to insufficient data on sNfL in CIDP. However, our sample size is similar to those generally used in the field of biomarker research in CIDP. All tests were 2-sided and confidence intervals (CIs) were reported as profile likelihood intervals.^34^

### Data availability

The anonymized data that support the findings of this study are available on reasonable request from the corresponding author, subject to local and European regulations.

## Results

### Demographic and clinical characteristics of the CIDP cohort

76 patients with CIDP were included with a mean (SD) age of 61.5 (11.7) years. All patients were of non-Hispanic Caucasian descent and 11/76 (14.5%) were female. 2/76 patients were diagnosed with another neurological disease (suspected Alzheimer’s disease) during follow-up, with symptom onset, respectively, 3 and 4 years after serum sampling. Cohort demographics and clinical features are summarized in Table 1. Information regarding 1-year disease progression was available for 71/76 patients (93.4%) while information regarding therapy response was available for all patients.

**Table 1:** Demographic and clinical characteristics of the CIDP cohort

Variable	*N* = 76
Age at time of sampling, mean (SD), years	61.5 (11.7)
Female, *n* (%)	11 (14.5)
Timespan between self-reported symptom onset and time of sampling, median (IQR), years	6.0 (6.9)
Time in follow-up at University Hospitals Leuven NMRC at last consult, median (IQR), years	6.9 (6.7)
Timespan between time of sampling and 1-year disease progression assessment, median (IQR), months	12.4 (1.7)
Timespan between time of sampling and therapy assessment at last consult, median (IQR), years	4.5 (4.2)
EFNS/PNS diagnostic criteria, *n* (%)	
Definite	63 (82.9)
Probable	13 (17.1)
Phenotype, *n* (%)	
Classic CIDP	58 (76.3)
Atypical CIDP	18 (23.7)
Acute onset	6
DADS	3
MADSAM	8
Pure sensory	1
Incidence of neurodegenerative disorders during follow-up, *n* (%)	
Suspected Alzheimer’s disease	2 (2.6)
None	74 (97.4)
1-Year disease progression, *n* (%)	
Progressors	16 (21)
Non-progressors	55 (72.4)
Unknown (missing data)	5 (6.6)
Therapy response over time, *n* (%)	
Responders	55 (72.4)
Non-responders	21 (27.6)
MRC sum-score at time of sampling, median (IQR)^a^	77 (6)
sNfL, median (IQR), pg/ml	28.3 (26.4)

DADS = distal acquired demyelinating symmetric neuropathy; EFNS/PNS = European Federation of Neurological Societies/Peripheral Nerve Society; MADSAM = multifocal acquired demyelinating sensory and motor neuropathy; NMRC = NeuroMuscular Reference Center.

a Available for 73/76 patients.

### Associations between sNfL and demographic and clinical characteristics

The median cohort sNfL was 28.3 pg/ml [interquartile range (IQR), 26.4 pg/l] with 18 patients having sNfL levels below the LLOQ (range, 1–15 pg/ml). sNfL was correlated with age at time of sampling, but not with disease duration, sex, CIDP phenotype or baseline MRC sum-score (Table 2). A multivariable model including demographics and baseline MRC sum-score confirmed the correlation between sNfL and age (Table 2).

**Table 2:** Associations between sNfL levels and demographics

Variable	sNfL, median (IQR), pg/ml	Univariate models			**Multivariable model** (MRC sum-score at time of sampling)			**Multivariable model** (1-year change in MRC sum-score)		
		β	95% CI	*P*	β	95% CI	*P*	β	95% CI	*P*
Age, years	–	1.183	0.822 to 1.545	<0.001	1.251	0.865 to 1.638	<0.001	1.268	0.862 to 1.674	<0.001
Sex										
Female (11)	24.6 (42.0)	–	–	–	–	–	–	–	–	–
Male (65)	28.3 (26.5)	3.665	−11.256 to 18.586	0.63	−5.571	−17.733 to 6.592	0.36	−5.498	−17.940 to 6.941	0.38
Disease duration, years	–	0.717	−0.199 to 1.634	0.12	−0.156	−0.943 to 0.632	0.70	−0.105	−0.906 to 0.696	0.79
CIDP Phenotype										
Typical (58)	26.3 (26.5)	–	–	–	–	–	–	–	–	–
Atypical (18)	38.7 (46.3)	6.195	−6.092 to 18.482	0.32	6.263	−3.768 to 16.294	0.22	5.462	−4.925 to 15.850	0.30
MRC sum−score at time of sampling^a^	–	−0.484	−1.082 to 0.115	0.11	−0.426	−0.910 to 0.058	0.08	–	–	–
1-Year change in MRC sum-score^b^	–	−0.642	−1.622 to 0.337	0.20	–	–	–	−0.933	−1.741 to −0.124	0.02

The number of patients for each category is indicated in parenthesis.

a MRC sum-score at time of sampling was available for 73/76 patients.

b One-year change in MRC sum-score information was available for 71/76 patients.

### sNfL as predictor of 1-year disease progression on MRC sum-score

Patients were classified as 1-year disease progressors when compared to baseline MRC sum-score, a decrease of four or more points was observed one year after sampling. Data regarding disease progression were missing for 5/76 patients and these patients were excluded from statistical analyses. 55/71 (77.5%) patients were classified as non-progressors with a median (IQR) change in MRC sum-score of 0 (2) points while 16/71 (22.5%) patients were classified as disease progressors with a median (IQR) decrease in MRC sum-score of six (2.25) points. sNfL levels were significantly increased in disease progressors compared to non-progressors [respectively median (IQR) sNfL of 48.7 (45.8) and 24.6 (28.5) pg/ml; *P* = 0.001]. A multivariable linear regression model also demonstrated a significant association between sNfL levels and the 1-year change in MRC sum-score (β, −0.933; 95% CI, −1.741 to −0.124; *P* = 0.02; Table 2).

sNfL as a continuous variable was found to be significantly associated with 1-year disease progression, both in univariate [odds ratio (OR), 1.049; 95% CI, 1.022–1.084; *P* = 0.001] and multivariable logistic regression models (OR, 1.097; 95% CI, 1.045–1.169*; P* = 0.001) (Table 3). When sNfL was included in the logistic regression models as binary variable, with patients having sNfL levels above the median cohort sNfL (28.3 pg/ml) being stratified as having ‘high’ sNfL, high sNfL levels were associated with strongly increased odds of 1-year disease progression (univariate: OR, 5.597; 95% CI, 1.590–26.457; *P* = 0.01; multivariable: OR, 6.572; 95% CI, 1.495–39.702; *P* = 0.02) (Table 3). Age and disease duration were also significantly associated with 1-year disease progression in the multivariable continuous sNfL logistic regression model, but not in the multivariable binary sNfL logistic regression model (Table 3).

**Table 3:** Logistic regression models for sNfL as predictor of 1-year disease progression

Variable	Univariate models			**Multivariable model** **(continuous sNfL)**			**Multivariable model** **(binary sNfL)**		
	OR	95% CI	*P*	OR	95% CI	*P*	OR	95% CI	*P*
Age, years	1.019	0.972–1.072	0.44	0.902	0.816–0.981	0.03	0.976	0.914–1.036	0.43
Disease duration, years	1.110	1.011–1.232	0.03	1.172	1.039–1.358	0.02	1.101	0.997–1.234	0.07
Phenotype, atypical	1.629	0.444–5.480	0.44	0.607	0.108–2.854	0.55	1.075	0.243–4.185	0.92
Sex, male	0.738	0.182–3.724	0.68	0.739	0.138–4.439	0.73	0.629	0.131–3.496	0.57
sNfL, pg/ml	1.049	1.022–1.084	0.001	1.097	1.045–1.169	0.001	–	–	–
High sNfL^a^	5.597	1.590–26.457	0.01	–	–	–	6.572	1.495–39.702	0.02

a Based on median cohort sNfL value (28.3 pg/ml), patients were binarily categorized as having ‘high’ or ‘low’ sNfL values. Data regarding disease progression were missing for 5/76 patients and these patients were not included in the analysis. 16/71 (22.5%) patients showed a decrease of four or more points on an 80-point MRC sum-score scale 1 year after sampling and were classified as disease progressors.

### sNfL as predictor of insufficient treatment response during follow-up

61/76 (80.3%) patients were receiving therapy at time of sampling, while for 14/76 (18.4%) patients intravenous immunoglobulin (13 patients) or plasma exchange (one patient) therapy was initiated within one year after sampling. One patient did not receive any therapy prior to sampling and during follow-up (>10 years) but remained stable without treatment. sNfL levels did not differ between patients receiving therapy at time of sampling versus those who did not [respectively median (IQR) sNfL 28.3 (25.6) and 26.3 (30.3) pg/ml; *P =* 0.98]. In total, baseline treatment to assess therapy response was intravenous immunoglobulin therapy for 61/76 patients (80.3%), plasma exchange for 6/76 patients (7.9%), a combination of intravenous immunoglobulins and steroids for 3/76 patients (3.9%) and a combination of intravenous immunoglobulins and azathioprine for 5/76 patients (6.6%). sNfL levels did not significantly differ between the different baseline treatment groups (Kruskal–Wallis *P =* 0.21). The median (IQR) time span over which therapy response was assessed was 4.5 (4.2) years with a minimum of one year. 21/76 patients (27.6%) were classified as non-responders when, compared to baseline therapy, a therapy switch was required throughout follow-up due to insufficient treatment effect (Table 1). One patient was switched from baseline therapy due to practical reasons of therapy administration but did respond to baseline treatment and was therefore classified as therapy responder. sNfL levels tended to be increased in non-responders but did not significantly differ from levels observed in therapy responders [respectively median (IQR) sNfL of 41.2 (33.3) and 25.2 (22.9) pg/ml; *P =*0.06].

sNfL as continuous variable was not significantly associated with insufficient treatment response (univariate model: OR, 1.021; 95% CI, 1.000–1.045; *P =* 0.06; multivariable: OR, 1.024; 95% CI, 0.997–1.054; *P =* 0.09) (Table 4). However, when sNfL was included as binary variable by stratifying patients according to median cohort sNfL (28.3 pg/ml), high sNfL levels were significantly associated with increased odds of insufficient treatment response, both in univariate (OR, 4.800; 95% CI, 1.622–16.442; *P =* 0.007) and multivariable (OR, 6.441; 95% CI, 1.749–29.357; *P =* 0.009) models (Table 4).

**Table 4:** Logistic regression models for sNfL as predictor of insufficient treatment response

Variable	Univariate models			**Multivariable model** **(continuous sNfL)**			**Multivariable model** **(binary sNfL)**		
	OR	95% CI	*P*	OR	95% CI	*P*	OR	95% CI	*P*
Age, years	1.018	0.975–1.065	0.42	0.987	0.928–1.046	0.66	0.979	0.918–1.036	0.48
Disease duration, years	0.999	0.908–1.090	0.98	0.986	0.890–1.084	0.78	0.973	0.875–1.070	0.59
Phenotype, atypical	2.000	0.632–6.128	0.23	1.926	0.567–6.372	0.28	1.753	0.475–6.197	0.39
Sex, male	4.444	0.771–84.321	0.17	4.871	0.792–94.954	0.15	5.212	0.828–102.20	0.14
sNfL, pg/ml	1.021	1.000–1.045	0.06	1.024	0.997–1.054	0.09	–	–	–
High sNfL^a^	4.800	1.622–16.442	0.007	–	–	–	6.441	1.749–29.357	0.009

a Based on median cohort sNfL (28.3 pg/ml) patients were binarily categorized as having ‘high’ or ‘low’ sNfL values. 21/76 (27.6%) patients required therapy switch during follow-up due to insufficient treatment response.

### sNfL as predictor of 1-year disease progression and/or therapy non-response during follow-up

As sNfL, in independent analyses, was associated with both 1-year disease progression and therapy response during follow-up, we next evaluated in a combined approach analysis whether sNfL levels could also differentiate patients who demonstrated 1-year disease progression, therapy non-response during follow-up or both from patients who remained stable on MRC sum-score while also responding to baseline therapy (i.e. responding non-progressors). 5/76 patients were excluded from analysis due to missing data regarding disease progression. 30/71 patients (42.3%) were assigned to the disease progressor and/or therapy non-responder group (consisting of 11 1-year disease progressors, 14 therapy non-responders and 5 non-responding disease progressors) while 41/71 patients (57.7%) remained stable on MRC sum-score while also responding to baseline therapy. sNfL levels were higher in the disease progressors and/or non-responders group than in patients who remained stable while responding to baseline therapy [respectively median (IQR) sNfL of 41.0 (27.7) and 20.3 (28.3) pg/ml); *P =* 0.006].

sNfL levels were significantly associated with demonstrating 1-year disease progression and/or therapy non-response (univariate: OR, 1.033; 95% CI 1.010–1.060; *P =* 0.008; multivariable: OR 1.051; 95% CI, 1.018–1.093; *P =* 0.005) (Table 5). When sNfL was included as binary variable, with sNfL levels above the median cohort sNfL (28.3 pg/ml) being stratified as ‘high’ sNfL, high sNfL levels corresponded with greatly increased odds of showing 1-year disease progression and/or therapy non-response (univariate: OR, 6.337; 95% CI, 2.276–19.469; *P* < 0.001; multivariable: OR, 10.138; 95% CI, 2.801–46.404; *P =* 0.001) (Table 5).

**Table 5:** Logistic regression models for sNfL as predictor for 1-year disease progression and/or therapy non-response during follow-up

Variable	Univariate models			**Multivariable model** **(continuous sNfL)**			**Multivariable model** **(binary sNfL)**		
	OR	95% CI	*P*	OR	95% CI	*P*	OR	95% CI	*P*
Age, years	1.019	0.978–1.063	0.38	0.948	0.884–1.007	0.10	0.955	0.890–1.012	0.15
Disease duration, years	1.070	0.985–1.174	0.12	1.088	0.985–1.220	0.12	1.071	0.971–1.198	0.20
Phenotype, atypical	1.293	0.425–3.899	0.65	0.805	0.209–2.837	0.74	0.892	0.229–3.275	0.86
Sex, male	2.182	0.569–10.700	0.28	2.899	0.667–15.771	0.18	2.632	0.595–14.477	0.22
sNfL, pg/ml	1.033	1.010–1.060	0.008	1.051	1.018–1.093	0.005	–	–	–
High sNfL^a^	6.337	2.276–19.469	<0.001	–	–	–	10.138	2.801–46.404	0.001

a Based on median cohort sNfL (28.3 pg/ml) patients were binarily categorized as having ‘high’ or ‘low’ sNfL values. Data regarding disease progression were missing for 5/76 patients and these patients were not included in the analysis. 30/71 patients (42.3%) demonstrated 1-year disease progression and/or therapy non-response during follow-up.

## Discussion

In recent years, the call for biomarkers to better predict disease progression or therapy response in CIDP has been increasing.^35–37^ While progress has been made with the discovery of paranodal autoantibodies associated with more severe disease course and poor response to intravenous immunoglobulins, these markers have only been described in a small subset (<15%) of patients.^6–8^ For the larger general CIDP population, prognostic serum-based biomarkers still present an unmet need. Recently, studies reported elevated sNfL levels in patients with peripheral neuropathies and some of these studies demonstrated significant cross-sectional associations between sNfL and impairment (including in CIDP), hinting at the idea of sNfL as individual prognostic marker in these disorders.^19–23^ In current study, we investigated in 76 patients with CIDP the association of baseline sNfL levels with 1-year disease progression and therapy response during follow-up.

Median sNfL levels in our cohort were measured to be 28.3 pg/ml, showing a mild increase in comparison to previously published sNfL levels of healthy and non-neurodegenerative controls (<10 pg/ml)^38^^,^^39^ while demonstrating similarity to a previously reported median of 27.2 pg/ml in 24 patients with CIDP receiving immunomodulatory therapy at time of sampling.^19^ We demonstrated that sNfL levels were associated with 1-year disease progression, which was in contrast to van Lieverloo et al.^19^ who did not observe associations between sNfL and change in impairment in patients in whom therapy was initiated at start of study. This discrepancy could possibly be explained through differences in study design: while we defined disease progression as a decrease of at least four points on an 80-point MRC sum-score scale over a 1-year period, van Lieverloo et al.^19^ measured impairment by various measurements including a 60-point MRC sum-score scale over a shorter period of six months. As the ‘standard’ 60-point scale has been shown to be less sensitive to changes in patients with only mild or distal CIDP,^35^ it is possible that the use of the 80-point scale—which assesses two additional distal muscle groups—enabled us to detect smaller changes in impairment. Another possible explanation might be situated in inclusion criteria: while in the study of van Lieverloo et al. most patients in whom no association between sNfL and change in impairment was found were newly diagnosed and treatment naive, the majority of our patients (61/76) were already receiving therapy at time of sampling. Since so far predominantly the short-term efficacy of CIDP therapies has been established,^37^^,^^40^ with only a limited number of studies addressing long-term efficacy, it may be possible that treatment naive patients still experience larger treatment effects and thus less short-term disease progression than patients who have been receiving treatment for a longer period of time as previously has been described for multifocal motor neuropathy, another neuroinflammatory peripheral nerve disease.^41^ However, a study in which the association of sNfL with disease progression is examined over a long period of time in treatment naive patients would be required to confirm sNfL as predictor for disease progression.

van Lieverloo et al.^19^ previously reported an association between CIDP patients not responding to therapy and increased sNfL levels at time of therapy assessment but, in contrast to current study, the authors did not investigate the predictive value of sNfL for therapy response. Our results now demonstrate that patients with sNfL levels above the median cohort sNfL had increased odds of insufficient treatment response during follow-up. As such, sNfL may hold value in the clinical decision making of CIDP by identifying those patients who would eventually require treatment switch or who should immediately be started on more intensive treatment (e.g. combination therapy). In a combined approach analysis, we also showed that sNfL levels were able to differentiate patients showing either 1-year disease progression, therapy non-response during follow-up or both from patients remaining stable on MRC sum-score while responding to baseline therapy with sNfL levels above median cohort sNfL being associated with greatly increased odds of showing 1-year disease progression and/or therapy non-response. Hence, the results of this combined approach further substantiate the association of sNfL with 1-year disease progression and therapy response during follow-up. As such, our findings suggest that baseline sNfL measurements might be useful to identify those patients most likely to demonstrate 1-year disease progression or therapy non-response which could be of interest in clinical trial design (e.g. in patient recruitment). Nevertheless, large scale studies that follow patients over longer periods of time would be desirable to confirm our preliminary results. Moreover, prospective longitudinal studies would also be interesting to not only further confirm the value of single baseline sNfL measurements but also to investigate the value of multiple serial sNfL measurements in CIDP’s clinical practice.

During our study, we encountered several limitations worth noting: first, due to retrospective study design, the included cohort was rather heterogeneous with respect to demographic variables. While mostly unassociated with sNfL levels, we nevertheless tried to counter this heterogeneity by including these demographics in our multivariable models. Second, retrospective design also implied that we could only include patients for whom residual material was available, which not only limited our cohort size but also affected cohort characteristics. For example, most patients for whom samples were stored were patients who were already receiving therapy at time of sampling (61/76). Also, it was observed that the earliest available sample stored in the biobank (i.e. the sample used for current study) often did not correspond to the ‘true’ baseline sample (i.e. taken at diagnosis or symptom onset) as most patients were already experiencing symptoms for multiple years at time of sampling (Table 1). However, other large scale studies that investigated the prognostic value of sNfL have previously also used similar approaches in which patients who were already receiving therapy at baseline or who were already experiencing symptoms for multiple years at baseline were included.^17^^,^^18^^,^^25^ Nevertheless, prospective studies utilizing newly diagnosed and fully treatment naive cohorts would be very interesting to further elucidate the prognostic value of sNfL in CIDP. Another limitation of retrospective design was that we had to define disease progression based on impairment measurements for which retrospective data were available for most included patients (i.e. the MRC sum-score). Ideally, however, other better-established disability measurements for CIDP - such as for example the Inflammatory Neuropathy Cause and Treatment scale - would be utilized to define disease progression, but as these disability measurements have only been introduced in UZ Leuven clinical practice in more recent years, data regarding e.g. Inflammatory Neuropathy Cause and Treatment scores at time of sampling, were missing for most patients. Hence, this restricted our definition of disease progression to solely be based on the MRC sum-score. A final limitation was that during sNfL measurements, 18 samples were encountered with concentrations below the previously reported LLOQ of the electrochemiluminescence assay (15.6 pg/ml).^30^ While we opted to use the original concentrations in our statistical analyses as a previous study showed that this resulted in best model performances,^31^ we still recommend future studies to prevent this analytical difficulty by utilizing more sensitive techniques such as single-molecule array assay.

In conclusion, our retrospective longitudinal study showed that in CIDP sNfL was associated with both 1-year disease progression on MRC sum-score and therapy response during follow-up, further hinting at the idea of sNfL as potential candidate prognostic biomarker in peripheral nerve disorders. However, further prospective studies are needed to confirm these preliminary results.

## Funding

J.G. and M.D.S. have PhD-fellowships of the Research Foundation—Flanders (respectively, 1191420N and 11E6319N). M.D.S. also received a grant of the Rotary’s ‘Espoir en Tête—Hoofdzaak er is Hoop’. K.P. received a start-up grant of the Group of Biomedical Sciences KU Leuven and holds a Clinical Research fund of the University Hospitals Leuven. P.V.D. holds a senior clinical investigatorship of the Research Foundation—Flanders and is supported by the ALS Liga België and the KU Leuven funds ‘een hart voor ALS’, ‘Laeversfonds voor ALS Onderzoek’ and the ‘Valéry Perrier Race against ALS Fund’.

## Competing interests

K.G.C. and P.V.D. are chairholders of the Emil von Behring Chair for Neuromuscular and Neurodegenerative Disorders by CSL Behring and are member of the European Reference Network for Rare Neuromuscular Diseases (ERN EURO-NMD) and of the European Reference Network for Rare Neurological Diseases (ERN-RND). K.G.C. received advisory board honoraria, research grants unrelated to the current study and travel reimbursement from Alnylam, Biogen, CSL Behring and Sanofi-Genzyme. J.G., M.D.S., X.B. and K.P. report no competing interests.

## Abbreviations

CI =: confidence interval
CIDP =: chronic inflammatory demyelinating polyneuropathy
IQR =: interquartile range
LLOQ =: lower limit of quantification
MRC =: Medical Research council
OR =: odds ratio
sNfL =: serum neurofilament light chain
